# *Legionella* Effector AnkX Disrupts Host Cell Endocytic Recycling in a Phosphocholination-Dependent Manner

**DOI:** 10.3389/fcimb.2017.00397

**Published:** 2017-09-08

**Authors:** Samual C. Allgood, Barbara P. Romero Dueñas, Rebecca R. Noll, Colleen Pike, Sean Lein, M. Ramona Neunuebel

**Affiliations:** Department of Biological Sciences, University of Delaware Newark, DE, United States

**Keywords:** *L. pneumophila*, AnkX, endocytic recycling, phagosome maturation, phosphocholination

## Abstract

The facultative intracellular bacterium *Legionella pneumophila* proliferates within amoebae and human alveolar macrophages, and it is the causative agent of Legionnaires' disease, a life-threatening pneumonia. Within host cells, *L. pneumophila* establishes a replicative haven by delivering numerous effector proteins into the host cytosol, many of which target membrane trafficking by manipulating the function of Rab GTPases. The *Legionella* effector AnkX is a phosphocholine transferase that covalently modifies host Rab1 and Rab35. However, a detailed understanding of the biological consequence of Rab GTPase phosphocholination remains elusive. Here, we broaden the understanding of AnkX function by presenting three lines of evidence that it interferes with host endocytic recycling. First, using immunogold transmission electron microscopy, we determined that GFP-tagged AnkX ectopically produced in mammalian cells localizes at the plasma membrane and tubular membrane compartments, sites consistent with targeting the endocytic recycling pathway. Furthermore, the C-terminal region of AnkX was responsible for association with the plasma membrane, and we determined that this region was also able to bind the phosphoinositide lipids PI(3)P and PI(4)P *in vitro*. Second, we observed that mCherry-AnkX co-localized with Rab35, a regulator of recycling endocytosis and with major histocompatibility class I protein (MHC-I), a key immunoregulatory protein whose recycling from and back to the plasma membrane is Rab35-dependent. Third, we report that during infection of macrophages, AnkX is responsible for the disruption of endocytic recycling of transferrin, and AnkX's phosphocholination activity is critical for this function. These results support the hypothesis that AnkX targets endocytic recycling during host cell infection. Finally, we have demonstrated that the phosphocholination activity of AnkX is also critical for inhibiting fusion of the *Legionella*-containing vacuole (LCV) with lysosomes.

## Introduction

*Legionella pneumophila* (strain Philadelphia-1, hereafter *Legionella*) is a Gram-negative, facultative intracellular bacterium that is regarded as an important cause of hospital- and community-acquired pneumonia (CDC, 2011; Viasus et al., 2013). In nature, *Legionella* proliferates within protozoa; however, human infection can occur following inhalation of contaminated aerosolized water droplets (Horwitz and Silverstein, 1980; Segal and Shuman, 1999). In the lung, alveolar macrophages engulf *Legionella* by phagocytosis, but are unable to degrade it through the usual process of phagosome maturation, which entails the sequential fusion of the phagosome with endocytic compartments and ultimately with the lysosome (Clemens et al., 2000). Instead, *Legionella* remains enclosed in a plasma membrane-derived compartment known as the *Legionella*-containing vacuole (LCV) that undergoes drastic remodeling into a compartment resembling the endoplasmic reticulum (Tilney et al., 2001). This process is dependent on the Dot/Icm specialized type IV secretion system (T4SS) that translocates over 300 bacterial effector proteins into the host cytosol (Berger and Isberg, 1993; Segal and Shuman, 1999; Luo and Isberg, 2004; Segal, 2013). Because of functional redundancy among *Legionella* effectors (O'Connor et al., 2011), the molecular strategies important for *Legionella*'s escape from phagolysosomal maturation are not yet well-understood. AnkX was first identified as one of several *Legionella* effector proteins that harbor ankyrin repeats (Pan et al., 2008). Mainly found in eukaryotes and some intracellular pathogens, ankyrin repeats are commonly involved in protein-protein interactions (Li et al., 2006), although for AnkX, whether these interactions involve any host components is unknown. The *Legionella* Δ*ankX* mutant is impaired in its ability to escape phagosome maturation, and more than 50% of the LCVs in Δ*ankX*–infected macrophage fuse with the lysosomal marker LAMP-1 (Pan et al., 2008). This is a dramatic effect considering that functional redundancy among *Legionella* effectors often masks phenotypes caused by the deletion of single genes. Although, existing data indicate that AnkX prevents microtubule-dependent vesicular transport (Pan et al., 2008), the precise mechanism through which AnkX inhibits LCV fusion with lysosomes has not yet been defined.

A subsequent study revealed that AnkX contains an N-terminal FIC domain (filamentation induced by cAMP; Roy and Mukherjee, 2009). The FIC domain is found in proteins from bacteria to humans and it typically catalyzes adenylylation or phosphocholination (Worby et al., 2009; Yarbrough et al., 2009; Mukherjee et al., 2011). AnkX's FIC domain catalyzes phosphocholination, which is the covalent addition of a phosphocholine moiety to a serine or a threonine residue of Rab GTPases (Mukherjee et al., 2011). Rab GTPases are small GTPases that function as molecular switches alternating between an active GTP-bound form and an inactive GDP-bound form (Stenmark, 2009). These proteins play central roles in defining the identity of membrane compartments and controlling membrane trafficking (Zerial and McBride, 2001). Growing evidence suggests that *Legionella* effector proteins tap into the host's membrane transport network by manipulating the activity of Rab GTPases either through mimicry of host regulatory proteins that control the nucleotide-bound state of Rab GTPases or by post-translational modification (Stein et al., 2012; Sherwood and Roy, 2013).

Rab1, a key regulator of ER-to-Golgi traffic, is recruited to the LCV early during infection, and multiple effectors are at work during infection to tightly control Rab1 activity (Machner and Isberg, 2006, 2007; Brombacher et al., 2009; Neunuebel et al., 2011, 2012; Tan and Luo, 2011; Mihai Gazdag et al., 2013; Mousnier et al., 2014). AnkX phosphocholinates inactive (GDP-bound) Rab1 which prevents its activation by guanine exchange factors (GEF) as well as its membrane extraction by the guanine dissociation inhibitor (GDI; Goody et al., 2012). In addition to Rab1, AnkX also phosphocholinates Rab35 and renders it unable to interact with Connecdenn, a protein that activates Rab35 by functioning as a GEF (Mukherjee et al., 2011). Despite these important biochemical observations, the biological functions of Rab GTPase phosphocholination have not yet been determined. In RAW 264.7 murine macrophages phosphocholination of Rab1 did not contribute to recruitment or retention of Rab1 on the LCV in this system (Hardiman and Roy, 2014). However, it is not yet known why AnkX targets Rab35 during infection.

Rab35 shares extensive sequence homology with Rab1; however, these Rab GTPases regulate distinct membrane transport pathways (Chua et al., 2010). Rab35 controls endocytic recycling of cargo between the plasma membrane and early endosomes (Klinkert and Echard, 2016) and has been implicated in phagosome maturation in both mammalian (Egami et al., 2011) and protozoan cells (Verma and Datta, 2017). An increasing number of bacterial pathogens have been reported to target endocytic recycling to promote their intracellular survival, including enterohemorrhagic *E. coli* (Furniss et al., 2016), uropathogenic *E. coli* (Dikshit et al., 2015), *Coxiella* (Larson and Heinzen, 2017), and *Chlamydia* (Ouellette and Carabeo, 2010; Larson and Heinzen, 2017). Given the known cellular functions of Rab35, we hypothesized that by targeting Rab35, AnkX would interfere with both endocytic recycling and phagosome maturation. Here, we demonstrate that *Legionella* disrupts endocytic recycling during macrophage infection. Moreover, we show that AnkX plays a significant role in disrupting endocytic recycling, and we reveal that its phosphocholination activity is critical for this function. We also found that in the absence of its phosphocholination activity, AnkX was unable to efficiently prevent acquisition of lysosomal markers on the LCV. We conjecture that AnkX interferes with phagosome maturation by disrupting endocytic recycling.

## Results

### AnkX localizes to the plasma membrane and peripheral endosomes

**Figure 1:**
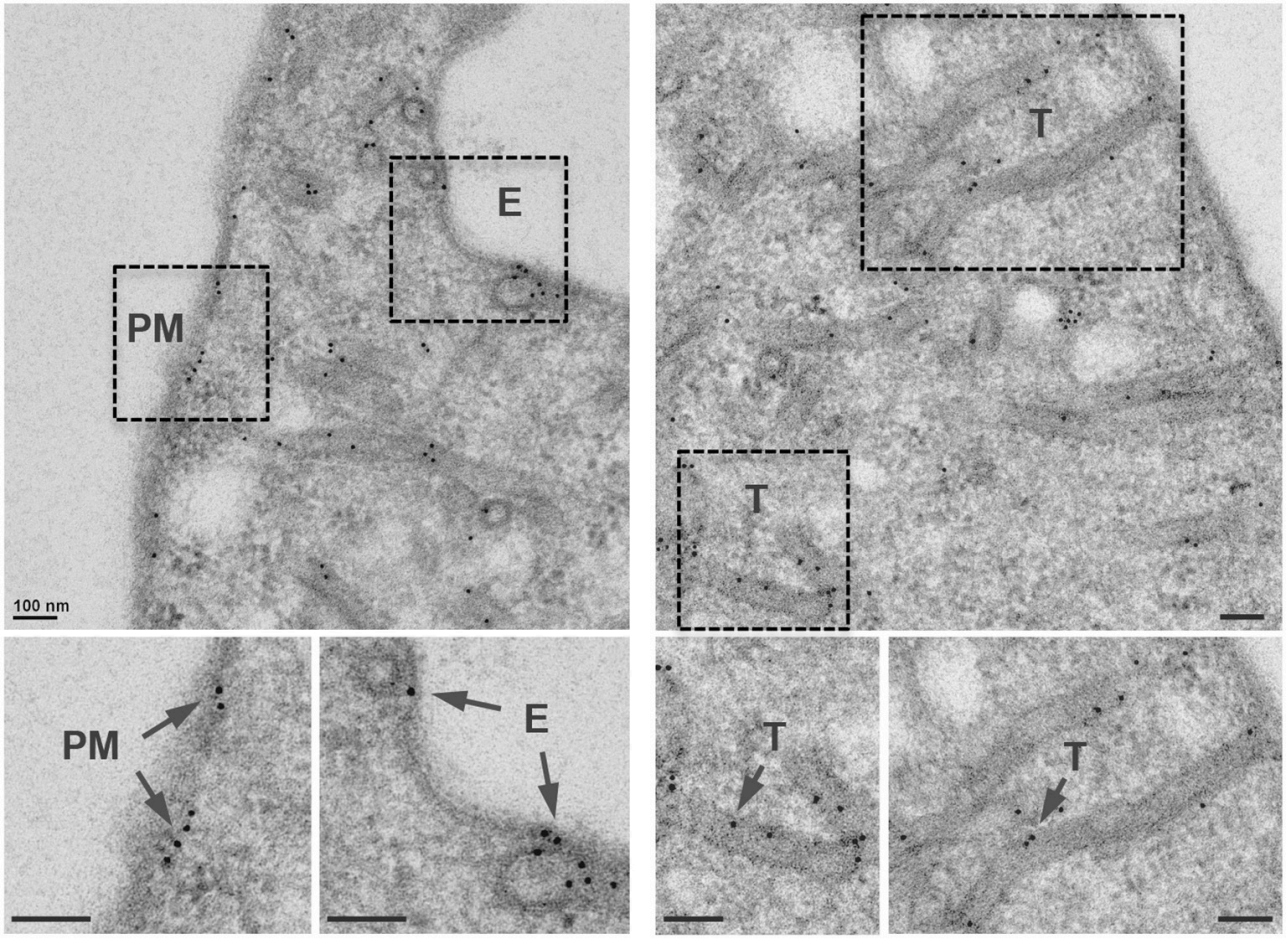
AnkX localizes to the plasma membrane, nascent endosomes, and tubular compartments. Representative TEM images showing immunogold localization of GFP-AnkX in COS-1 cells. Fixed cells were stained with a polyclonal rabbit anti-GFP antibody. Areas highlighted by rectangles (dashed line) on the top panels are magnified in the bottom panels. Arrows indicate immunogold labeled GFP-AnkX localized to endosomes (E), plasma membrane (PM), or tubules (T). Scale bar is 100 nm.

A previous study reported that AnkX was distributed in a punctate pattern when produced ectopically in mammalian cells (Pan et al., 2008). Immunostaining with an early endosome marker showed that GFP tagged AnkX did not co-localize with early endosomes (Mukherjee et al., 2011). We hypothesized that AnkX's cellular destination may be, at least in part, determined by the localization of its enzymatic substrates, Rab1 and Rab35. However, AnkX did not localize at the Golgi where Rab1 is predominantly found (Mukherjee et al., 2011), and we reasoned that perhaps AnkX is co-opted at cellular locations where Rab35 is present, namely the plasma membrane and endosomes. We fused AnkX to an N-terminal GFP tag and analyzed its subcellular localization within transiently transfected COS-1 cells using immunogold transmission electron microscopy. We determined that GFP-AnkX was associated with the plasma membrane, nascent endosomes, and tubular compartments at the periphery of the cell, reminiscent of the subcellular localization of recycling endosomes (Figure 1, Supplementary Figures 1, 2). These membrane compartments shuttle cellular components to the plasma membrane and commonly assume a tubular morphology (van Ijzendoorn, 2006).

### The C-terminal half of AnkX associates with plasma membrane

**Figure 2:**
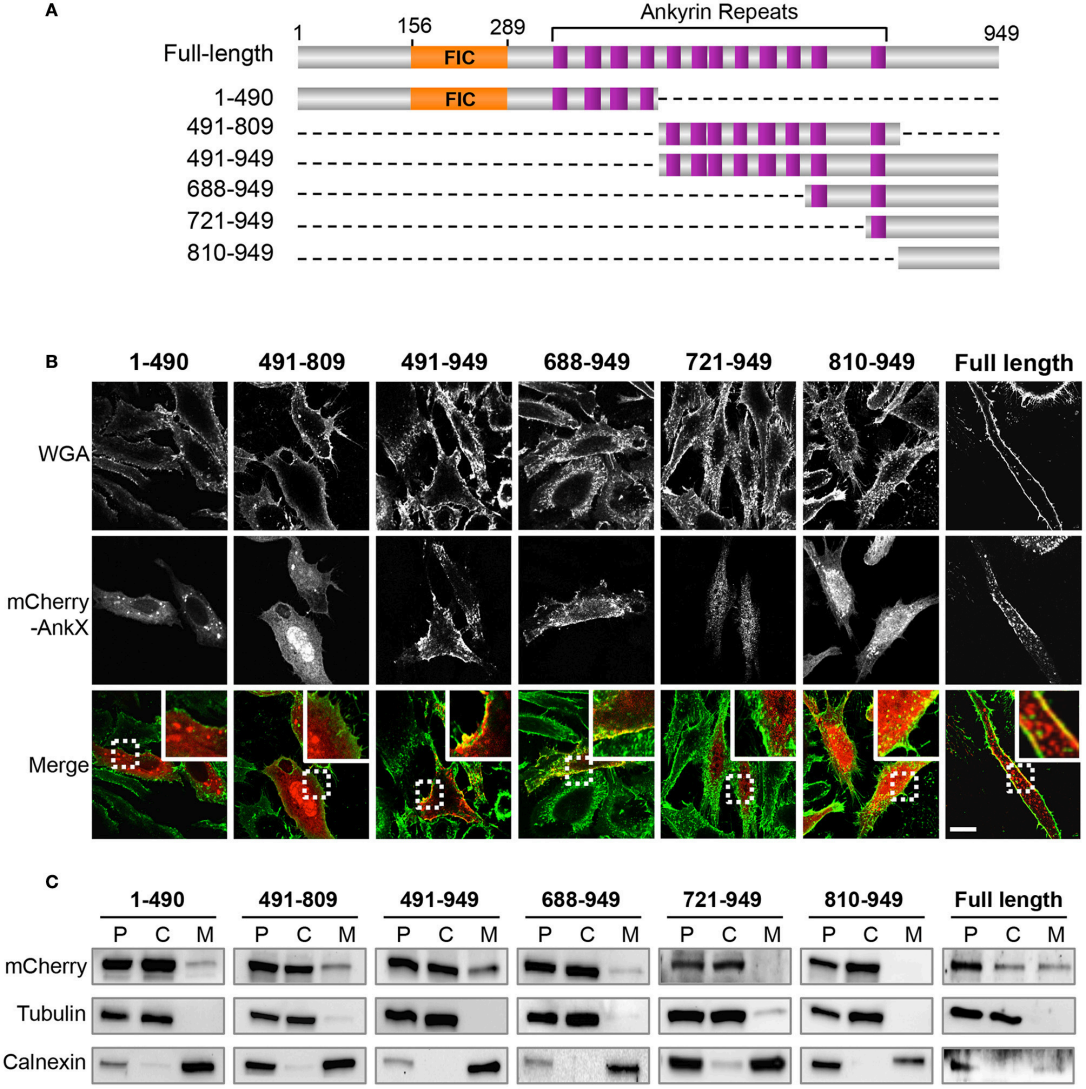
AnkX domain mapping. **(A)** Schematic of AnkX domain variants (The FIC domain, orange; 12 ankyrin repeats, purple; dashed lines, deleted region). **(B)** Subcellular localization of AnkX fragments. HeLa cells transiently producing mCherry-AnkX or mCherry-AnkX variants were incubated with the plasma membrane dye WGA-488 post-fixation and intracellular localization was determined by confocal microscopy. Images show one z-slice of the confocal stack. **(C)** Cellular fraction of HEK293T cells producing mCherry-AnkX or mCherry-AnkX variants. The post-nuclear supernatant (P) was separated by ultracentrifugation into cytosolic (C) and membrane (M) fractions. The presence of mCherry-AnkX variants in each fraction was assessed by immunoblotting using a mCherry antibody. Tubulin and calnexin were used as markers for the cytosolic and membrane fractions, respectively.

We then investigated which region of AnkX was responsible for localization to the plasma membrane. To address this question we generated truncated variants of AnkX fused to an N-terminal mCherry tag as follows: AnkX1-490 (containing the FIC domain and 4 ankyrin repeats), AnkX688-949 (containing 2 ankyrin repeats), AnkX721-949 (containing 1 ankyrin repeat), AnkX491-809 and AnkX491-949 (containing the same 8 ankyrin repeats), and AnkX810-949 (containing no ankyrin repeats; Figure 2A). These fragments were selected based on secondary structure predictions and available structural information (Campanacci et al., 2013). To establish which of these fragments localize at the plasma membrane, we transiently transfected HeLa cells with constructs carrying the *mCherry*-fused *ankX* truncations or full length *ankX*. We then stained the cells with Wheat Germ Agglutinin, a carbohydrate-specific dye that marks the plasma membrane, and analyzed the localization of mCherry-tagged AnkX and AnkX fragments in relation to the plasma membrane (Figure 2B). As expected based on the TEM results, mCherry-AnkX co-localized with WGA at the plasma membrane. In singly transfected cells, we did observe that mCherry-AnkX was not uniformly distributed along the plasma membrane, and instead it assumed a rather patchy distribution, favoring one side of the cell (Supplementary Figure 4). In 5% of the cells, mCherry-AnkX did not appear to be present at the plasma membrane, at least not in levels detectable by confocal microscopy (Supplementary Figure 4). Among all other constructs visualized by confocal microscopy only cells producing mCherry-AnkX491-949 or mCherry-AnkX688-949 displayed fluorescence at the plasma membrane. HeLa cells producing either mCherry-AnkX721-949 or mCherry-AnkX810-949 displayed fluorescence predominantly in the cytosol, although the latter also displayed lower signal in the nucleus. The FIC domain-containing mCherry-AnkX1-490 variant was dispersed throughout the cytosol and was also present on vesicular compartments, whereas mCherry-AnkX491-809 was present in the cytosol, and, surprisingly also in the nucleus. Therefore, the C-terminal half of AnkX possesses properties that provide AnkX with the ability to bind the plasma membrane, but also to bind nuclear components. To further analyze the cellular distribution of mCherry-tagged AnkX variants, we then performed cellular fractionation studies using HEK293T cells producing these protein variants. We chose to employ HEK293T cells for this approach due to their increased transfection efficiency with AnkX-derived constructs. Cellular fractionation studies confirmed that mCherry-AnkX, mCherry-AnkX491-949, and mCherry-AnkX688-949 were membrane-associated (Figure 2C). A lighter band was detected for mCherry-AnkX688-949 in the membrane fraction compared to cells producing mCherry-AnkX491-949. It is possible that this shortened fragment had a weaker interaction with the plasma membrane that was disrupted during ultracentrifugation. As expected based on our confocal microscopy results, mCherry-AnkX721-949 and mCherry-AnkX810-949 were found in the cytosolic fraction. Both mCherry-AnkX1-490 and mCherry-AnkX491-809 were present in the cytosolic and membrane fractions indicating that these fragments are potentially also present on endosomes.

**Figure 3:**
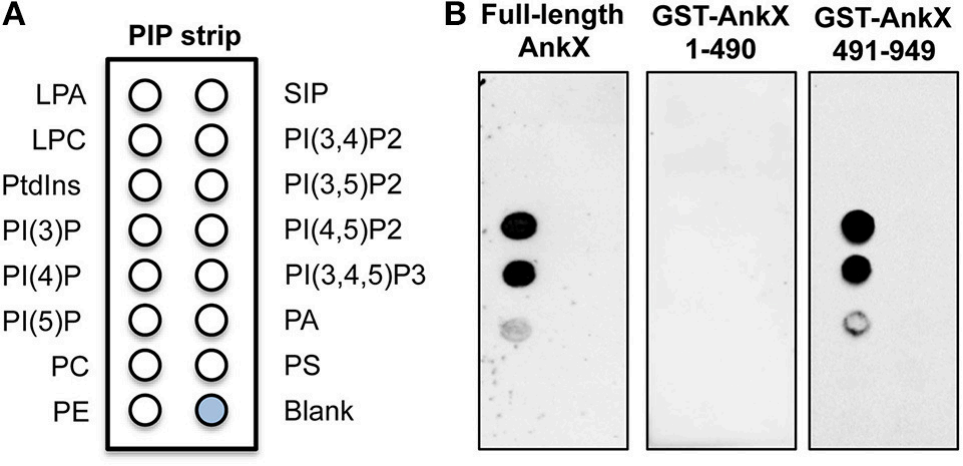
AnkX interacts with phosphoinositide lipids via its C-terminal region. **(A)** Schematic of lipids spotted on the phosphoinositide strip (LPA, lysophosphatidic acid; LPC, lysophosphocholine; PtdIns, phosphatidylinositol; PE, phosphatidylethanolamine; PC, phosphatidylcholine; SIP, sphingosine-1-phosphate; P, phosphate; P2, biphosphate; P3, triphosphate; PA, phosphatidic acid; PS, phosphatidylserine). **(B)** Protein-lipid overlay assay reveals that a C-terminal fragment of AnkX preferentially binds PI(3)P and PI(4)P. Untagged full-length AnkX was detected with an AnkX antibody and a GST antibody was used to detect the GST-tagged truncated versions of AnkX.

To identify the molecular mechanism underlying AnkX localization at the plasma membrane, we analyzed AnkX's ability to bind phosphoinositide lipids. Multiple *Legionella* effectors were reported to specifically recognize and bind phosphoinositide lipids (Hilbi et al., 2011). Most of the phosphoinositide binding effectors identified thus far bind PI(4)P and/or PI(3)P (Hilbi et al., 2011). Given that the LCV is enriched in PI(4)P, phosphoinositide binding is thought to serve as an anchoring mechanism for *Legionella* effectors that attach to the cytosolic surface of the LCV after translocation into the host cell. Additionally, this mechanism can facilitate targeting of particular host membrane compartments (Pizarro-Cerda et al., 2014). To evaluate AnkX's ability to bind phosphoinositide lipids, we performed a protein-lipid overlay assay using purified GST-AnkX and a commercially available nitrocellulose membrane spotted with phosphatidylinositol and all seven phosphoinositide species (Figure 3A). Through this approach we determined that AnkX bound mainly PI(3)P and PI(4)P (Figure 3B). To identify which region of AnkX was responsible for phosphoinositide binding, we generated two GST-tagged AnkX variants: GST-AnkX1-490 and GST-AnkX491-949 (Supplementary Figure 3). Our protein-lipid overlay assay showed that the C-terminal region of AnkX was capable of phosphoinositide binding while the N-terminal region was not (Figure 3B). We concluded that the C-terminal half of AnkX harbors the elements necessary to mediate association with the plasma membrane.

### AnkX targets Rab35-positive compartments

**Figure 4:**
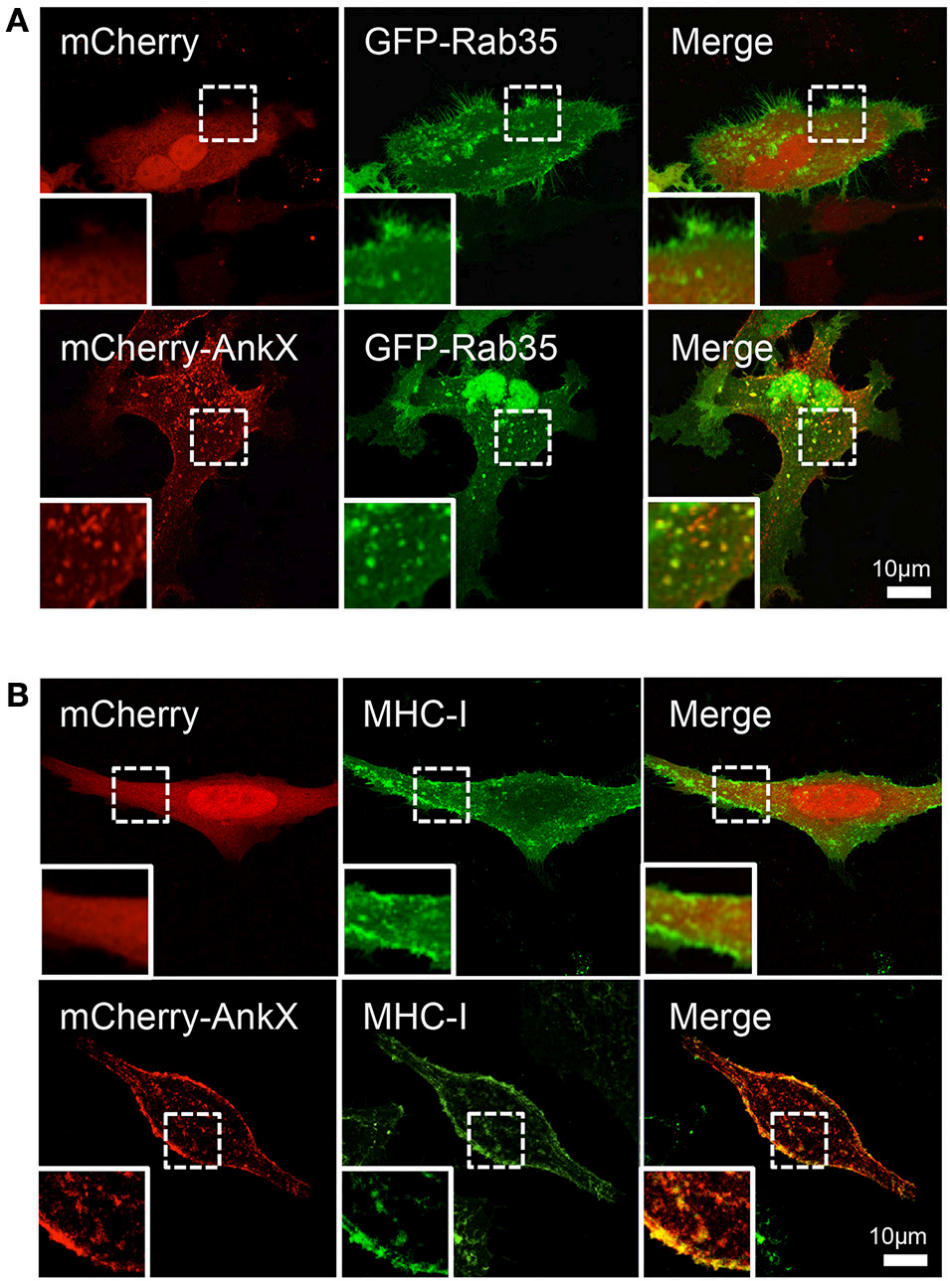
AnkX co-localizes with Rab35 and MHC-I, a Rab35-dependent cargo. **(A)** Confocal images of HeLa cells co-transfected with plasmids encoding mCherry and GFP-Rab35 or plasmids encoding mCherry-AnkX and GFP-Rab35. Insets show magnified view of an area of interest. Bottom panel insets highlight an area where fluorescent signal from the two proteins co-occurred on intracellular vacuolar compartments. **(B)** HeLa cells ectopically producing mCherry or mCherry-AnkX were allowed to internalize monoclonal fluorescently labeled MHC-I antibodies for 30 min, followed by an acid wash. Bottom panel insets highlight the compartments where mCherry-AnkX co-occurred with internalized MHC-I.

AnkX's localization at the plasma membrane and its ability to phosphocholinate Rab35 suggested that AnkX could target Rab35-positive recycling endosomes. To determine whether AnkX associates with these endosomes, we transiently co-transfected HeLa cells with a plasmid encoding GFP-Rab35 and a plasmid encoding either mCherry-AnkX or mCherry as a control. By confocal microscopy, we observed that the GFP-Rab35 signal was present in the cytosol, membrane compartments, the plasma membrane, and in some cells the nucleus (Figure 4A); nuclear localization of GFP-Rab35 has previously been observed, but this is believed to be caused by nuclear translocation of GFP alone (Kouranti et al., 2006). In agreement with our electron microscopy data, mCherry-AnkX was found to be present at the plasma membrane and on intracellular membrane compartments. When the two proteins were co-produced they co-localized on vacuolar compartments that resembled enlarged endosomes. Similar structures were observed in cells producing GFP-Rab35 in the absence of AnkX (Figure 4A).

Rab35 regulates recycling of protein cargo between the plasma membrane and endosomes, and therefore, we next sought to determine whether AnkX targets Rab35-positive recycling endosomes. The major histocompatibility Class I (MHC-I) enters cells through clathrin-independent endocytosis and then either returns back to the plasma membrane via recycling tubules or is degraded by lysosomes (Caplan et al., 2002). Loss of Rab35 activity has been shown to inhibit MHC-I recycling, indicating that its recycling back to the plasma membrane is, at least in part, dependent on Rab35 (Allaire et al., 2010). To determine whether AnkX targets recycling tubules that are carrying MHC-I, we performed an antibody uptake assay, whereby HeLa cells producing mCherry or mCherry-AnkX were incubated with MHC-I antibody conjugated with Alexa 488 fluorophore to mark endosomes that internalize MHC-I. Using confocal microscopy, we observed that mCherry-AnkX co-localized extensively with MHC-I at the plasma membrane and on tubular compartments (Figure 4B). These results support the hypothesis that AnkX targets Rab35-positive recycling endosomes in human cells.

### AnkX disrupts endocytic recycling during macrophage infection

**Figure 5:**
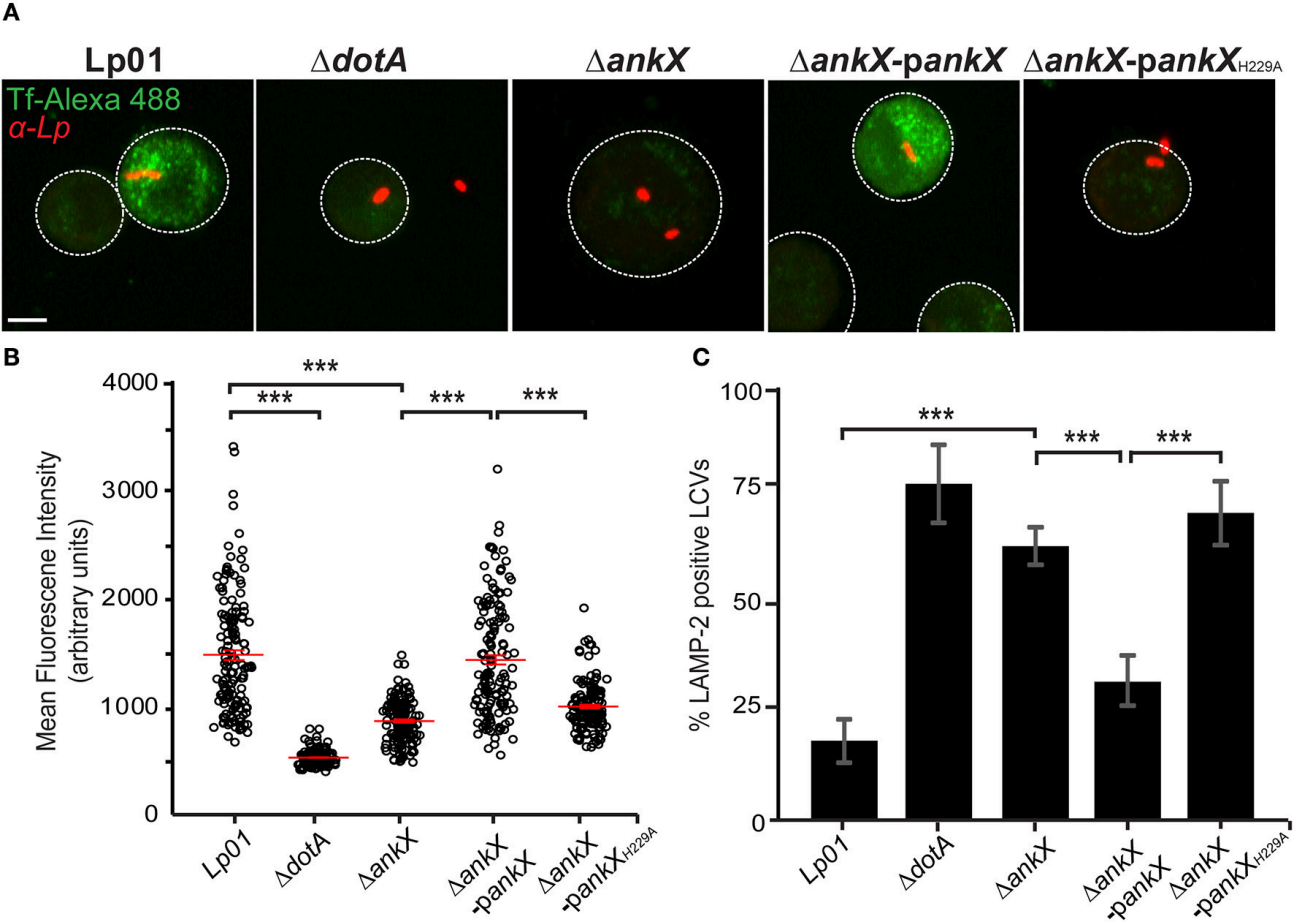
*Legionella* blocks host endocytic recycling and prevents accumulation of lysosomal markers on the LCV in a phosphocholination-dependent manner. **(A)** Representative images of human transferrin-Alexa 488 fluorescent signal in U937 cells infected with *Legionella* strains as labeled. At 1 h post-infection, U937 cells were incubated with transferrin-Alexa 488 for 60 min, then washed and incubated with unlabeled transferrin for 30 min. Cells were fixed and immunostained with anti-*Legionella* antibodies and visualized by confocal microscopy; scale bar, 5μm. **(B)** The graph displays the amount of fluorescence retained by infected U937 cells. The mean and standard error of the mean from three independent experiments are indicated for each condition. Statistical significance was determined by one-way ANOVA followed by Tukey Kramer *post-hoc* test. The asterisks denote a *p* < 0.001. **(C)** Quantification of LAMP-2 positive LCVs in U937 cells infected with various strains of *Legionella*. Bar graph displays the percentage of LVCs that were LAMP-2 positive at 2 h post-infection. Statistical significance was determined using the Student's *t*-test with a *p*-value cut off of < 0.05. The asterisks denote a *p* < 0.001.

A series of biochemical approaches have shown that phosphocholination prevents activation of Rab35. Therefore, we hypothesized that during infection AnkX would have the same effect, and thus disrupt the normal dynamics of recycling endosomes. To determine whether AnkX affects endocytic recycling during macrophage infection, we performed a transferrin-recycling assay. Transferrin is routinely used as a marker for endocytic recycling; it remains bound to its receptor throughout the recycling pathway and is released outside the cell upon returning to the cell surface (Mellman, 1996). U937 macrophages were infected with Lp01, Lp01Δ*dotA*, Lp01Δ*ankX*, Lp01Δ*ankX* complemented with a plasmid carrying wild-type *ankX*, or Lp01Δ*ankX* complemented with a plasmid carrying *ankX*_H229A_, a point mutant in the catalytic motif of the FIC domain that renders the protein catalytically inactive (Mukherjee et al., 2011). At 1 h post-infection, macrophages were subjected to a pulse-chase sequence with fluorescently labeled transferrin followed by incubation with unlabeled transferrin (Figure 5A). After the pulse step, U937 cells retained comparable levels of transferrin regardless of which *Legionella* strain they were infected with (Supplementary Figure 6). We then quantified the amount of fluorescently labeled transferrin remaining after a 30-min chase with unlabeled transferrin (Figure 5B). Macrophages infected with Lp01 displayed the highest level of fluorescence, indicating increased retention of transferrin within these cells. This result supports the idea that endocytic recycling of transferrin was blocked at an early stage of infection. In contrast, macrophages infected with the Lp01Δ*dotA* translocation-deficient mutant showed the lowest fluorescence signal among the five conditions, indicating that endocytic recycling of transferrin was robust in the absence of translocated *Legionella* effectors. Macrophages infected with the Lp01Δ*ankX* strain displayed levels of fluorescence that were significantly lower than those of cells infected with Lp01, and, thus, in the absence of AnkX, labeled transferrin along with its receptor were recycled back to the plasma membrane. We also noted that, although significantly reduced in Lp01Δ*ankX*-infected cells, the amount of labeled transferrin was 1.6 times higher than that detected in macrophages infected with the Lp01Δ*dotA* mutant (Figure 5B). When macrophages were infected with the *ankX* complemented Lp01Δ*ankX* strain, retention of labeled transferrin increased to a level similar to that of the Lp01-infected cells. Interestingly, macrophage infection with the Lp01Δ*ankX* ectopically producing the catalytically inactive AnkX^H229A^ resulted in diminished retention of labeled transferrin compared to macrophages infected with the Lp01Δ*ankX* strain producing active AnkX (Figure 5B). This change was not attributable to a difference in the production of AnkX or AnkX_H229A_ within the Lp01Δ*ankX* background since we verified that the two proteins were produced at comparable levels prior to macrophage infection (Supplementary Figure 5). Based on these results we concluded that the phosphocholination activity of AnkX was responsible for disruption of endocytic recycling. Notably, transferrin was not present at the LCV, consistent with previous findings that this compartment lacks transferrin (Joshi et al., 2001).

### Phosphocholination activity of AnkX is required for avoidance of phagosome maturation

The Lp01Δ*ankX* mutant has previously been shown to be impaired in its ability to escape phagosome maturation early following macrophage infection (Pan et al., 2008). Our results above indicated that the phosphocholination activity of AnkX is important for exerting its biological function. Therefore, we hypothesized that phosphocholination could play a key role in the avoidance of phagolysosome maturation. To test this hypothesis, we infected U937 macrophages with Lp01, Lp01Δ*dotA*, Lp01Δ*ankX*, Lp01Δ*ankX* complemented with a plasmid carrying wild-type *ankX*, and Lp01Δ*ankX* complemented with a plasmid carrying *ankX*_H229A_. At 2 h post-infection, macrophages were fixed, immunostained with an antibody against the lysosomal marker LAMP-2, and quantified with regard to the number of LAMP-2 positive LCVs. U937 cells infected with wild-type displayed 18(±5)% of LAMP-2 positive vacuoles, whereas cells infected with Lp01Δ*dotA* and Lp01Δ*ankX* displayed 77.3(±9)% and 63(±4.3)% LAMP-2 positive vacuoles, respectively (Figure 5C). These results are in agreement with findings reported by Pan et al. (2008). In addition, we showed that when macrophages were infected with the Lp01Δ*ankX* strain complemented producing AnkX the percentage of LAMP-2 positive LCVs decreased to 31.6(±6)%. In contrast, infection with the Lp01Δ*ankX* strain complemented with catalytically inactive AnkX^H229A^ did not rescue the phenotype and showed that 70.6(±7.3)% LCVs were LAMP-2 positive, similar to the percentage observed in cells infected with the Lp01Δ*ankX* or the Lp01Δ*dotA* strains. Therefore, we concluded that AnkX's phosphocholination activity plays a critical role in avoidance of phagosome maturation.

## Discussion

In eukaryotic cells, membrane transport plays essential roles in regulating signaling, metabolism, immunity, and interactions with the extracellular environment, and therefore, it is a major target for intracellular pathogens. *Legionella* manipulates the host's membrane transport system by delivering effector proteins that hijack Rab GTPases, a large class of small GTPases (over 60 in humans) performing essential roles in membrane trafficking (Stenmark, 2009). Delineating membrane transport pathways crucial for *Legionella* survival is complicated by the functional redundancy among *Legionella* effectors, and usually deletion of individual *Legionella* genes encoding effectors that target Rab GTPases does not markedly affect bacterial survival (O'Connor et al., 2011). Despite this hurdle, a previous study showed that deletion of *ankX* alone significantly increased fusion of LCVs with lysosomes revealing that AnkX plays an important role in safeguarding the LCV from phagosome maturation (Pan et al., 2008). However, the mechanistic details of how AnkX is involved in this process are not precisely understood. Here we narrow in on the biological consequences of AnkX activity during macrophage infection and demonstrate that AnkX disrupts endocytic recycling and phagolysosome biogenesis in a phosphocholination-dependent manner.

The *Legionella* effector AnkX functions as a phosphocholine transferase, and of the six Rab GTPases previously tested, Rab1 and Rab35 were efficiently phosphocholinated by AnkX (Mukherjee et al., 2011). Although the biochemical details of Rab1 and Rab35 phosphocholination are well-understood, the biological consequences of Rab1 and Rab35 phosphocholination remain elusive. Rab1 is heavily recruited to the LCV early during infection (Kagan et al., 2004), however AnkX is not required for this process (Hardiman and Roy, 2014). The importance of Rab35 manipulation by *Legionella* effectors has not yet been explored. Rab35 regulates a number of cellular processes including endocytic recycling and cytokinesis, exosome release, phagocytosis, cell migration, immunological synapse formation and neurite outgrowth (Klinkert and Echard, 2016). Because it is involved in shuttling protein cargo between the plasma membrane and early endosomes through recycling endosomes, Rab35 localizes predominantly at the plasma membrane and on tubular recycling endosomes (Chua et al., 2010). Using electron microscopy, we found that GFP-tagged AnkX had a localization pattern similar to that of Rab35 (Figure 1). Moreover, in HeLa cells mCherry-AnkX co-localized with GFP-Rab35 on vacuolar compartments (Figure 4A). These compartments could be enlarged early, sorting, or recycling endosomes, where Rab35 is known to localize. Alternatively, they may be similar to vacuolar compartments that have been observed to form as a consequence of Rab35 inactivation (Kouranti et al., 2006). If this were the case the formation of these Rab35-positive vacuoles would likely be a consequence of AnkX's inhibitory effect on Rab35 activity. However, other indirect effects cannot be excluded until confirmation by experiments using mCherry-AnkX^H229A^.

We next asked whether association of AnkX with the plasma membrane was due to its interaction with Rab35. We addressed this question by determining the subcellular localization of mCherry-tagged AnkX fragments in mammalian cells in relation to the plasma membrane. Surprisingly, we found that the N-terminal AnkX fragment containing the FIC domain did not associate with the plasma membrane, indicating that targeting of Rab35 is not sufficient to stably recruit AnkX to the plasma membrane (Figure 2). However, mCherry-AnkX1-490 can still localize to intracellular vesicles. A potential explanation for this observation is that AnkX's substrate specificity is more relaxed allowing other Rab GTPases to be modified, and perhaps the C-terminal region required for binding the plasma membrane limits AnkX's activity to specific compartments. Further insight into AnkX's substrate preference could clarify this issue.

The AnkX491-949 fragment contained the elements necessary to mediate plasma membrane binding. We showed that this fragment was also able to bind PI(3)P and PI(4)P (Figure 3), although it is not yet clear if phosphoinositide binding alone is sufficient to mediate association with the plasma membrane. Furthermore, binding of multiple phosphoinositide species may suggest that AnkX has functional roles at several stages of infection since *Legionella* actively changes the phosphoinositide composition of the LCV (Weber et al., 2014). Within seconds following uptake of *Legionella* by amoebae, the early phagosome membrane displays PI(3,4,5)P, immediately followed by a rise in PI(3)P levels. Subsequently, the vacuolar membrane gradually loses these lipids and by 2 h post-infection becomes enriched in PI(4)P. PI(3)P- and PI(4)P-binding regions of *Legionella* effectors are thought to mediate anchoring to the LCV (Weber et al., 2006; Hilbi et al., 2011); they may also function in conjunction with Rab GTPase binding domains as a two-pronged mechanism to specifically target membrane compartments. The precise region of AnkX that recognizes PI(3)P and PI(4)P remains to be identified. Intriguingly, the AnkX688-949 fragment was present at both the plasma membrane and on endosomes, whereas the AnkX721-949 fragment missing just one ankyrin repeat lost its membrane localization (Figure 2B). It is possible, therefore, that this ankyrin repeat or other elements in this region mediates the binding. We hypothesize that a multifactorial mechanism regulates AnkX interaction with the plasma membrane, wherein both Rab35 and phosphoinositide binding are both important, but an additional component may be necessary to stabilize the interaction.

In COS-7 cells, a Rab35 siRNA knockdown causes enlargement of early endosomes (Allaire et al., 2010), and, intriguingly, heterologous production of AnkX in mammalian cells results in a similar phenotype (Mukherjee et al., 2011). Given AnkX's co-localization with Rab35 (Figure 4A) and its known inhibitory effect on Rab35 activity *in vitro*, we reasoned that AnkX would disrupt pathways regulated by this Rab GTPase. Rab35 has been implicated in the recycling of various cargo proteins back to the plasma membrane including the MHC-I, MHC-II, and the T-cell receptor (Patino-Lopez et al., 2008; Walseng et al., 2008; Allaire et al., 2010). Our MHC-I internalization and recycling assay showed that AnkX co-occurred with MHC-I, supporting the notion that AnkX targets Rab35-dependent endocytic recycling (Figure 4B). MHC-I is involved in presenting intracellular antigens to cytotoxic T cells such that cells infected with intracellular pathogens can be eliminated (Mantegazza et al., 2013). It is plausible that *Legionella* disrupts recycling of these receptors in order to modulate host immune responses during infection to prevent the death of its host cell.

In light of these findings, we hypothesized that AnkX manipulates endocytic recycling during infection of macrophages by targeting Rab35. Our data showed that, indeed, endocytic recycling of transferrin, a widely used marker for this transport pathway, is blocked early during macrophage infection (Figures 5A,B). The inhibitory effect was dependent on *Legionella* effectors since infection with a translocation-deficient mutant did not disrupt transferrin recycling. Infection with the Lp01 Δ*ankX* mutant showed a sharp decrease in accumulation of transferrin-positive vesicles, indicating that AnkX is largely responsible for disruption of endocytic recycling. This phenotype was reversed when the mutant was complemented with ectopically expressed *ankX*, but not when it was complemented with *ankX*_H229A_. The necessity of an enzymatically active AnkX highlighted the key role of phosphocholination in disruption of transferrin recycling. Importantly, this result implies that phosphocholination of Rab35, and perhaps other host proteins, leads to disruption of endocytic recycling. Notably, the absence of AnkX did not abrogate *Legionella*'s ability to block endocytic recycling. This observation supports the idea that other *Legionella* effectors may also interfere with endocytic recycling.

Targeting of endocytic recycling appears to be a common strategy for intracellular pathogens. For example, uropathogenic *E. coli* (UPEC) has been proposed to manipulate endosomal recycling and degradative lysosomal fusion by modulating Rab35 to survive within bladder epithelial cells (Dikshit et al., 2015), and enterohemorrhagic *E. coli* (EHEC) modulates Rab35 recycling by secretion of the effector protein EspG in order to prevent maturation of recycling endosomes during infection (Furniss et al., 2016). More recently, a high-content imaging study showed that transferrin uptake gradually increased throughout infection of HeLa cells with *Coxiella burnetii* or *Chlamydia trachomatis* suggesting that bacterial infection expanded the endosomal system to increase capacity for endocytic material (Larson and Heinzen, 2017). The *Shigella* effector protein, IpgD, is responsible for recruiting Rab11-positive vesicles to the invasion site to assist in the rupture of the *Shigella*-containing vacuole, a step required for bacterial escape into the cytosol (Mellouk et al., 2014).

An important question is why *Legionella* would manipulate transferrin recycling during infection. It is unlikely that the pathogen is attempting to transport iron to the LCV as *Legionella* genetically encodes and translocates its own iron-sequestering pathway, which has been shown to be important for infection (Joshi et al., 2001; Chatfield et al., 2012; Isaac et al., 2015). Instead, we propose that *Legionella* effectors target Rab35 to counteract phagosome maturation. Upon phagocytosis into macrophages, *Legionella* immediately prevents phagosome maturation, avoiding fusion of early endosomes with the LCV. Rab35 is involved in formation of the phagocytic cup in macrophages (Egami et al., 2011). Silencing of Rab35 by RNA interference drastically reduced the rate of FcγR-mediated phagocytosis in macrophages, revealing that Rab35 is involved in the early stage of phagocytosis. Interestingly, in *Entamoeba histolytica*, an intestinal protozoan parasite, Rab35 is not only required for phagocytic cup formation, but also for biogenesis of phagolysosomal compartments (Verma and Datta, 2017). In light of these results, we hypothesized that similar to UPEC, *Legionella* manipulates Rab35 to subvert both endosomal recycling and degradative lysosomal fusion. Therefore, we anticipated that AnkX's phosphocholination activity would be crucial for its previously established ability to prevent fusion of lysosomes with the LCV. Our results confirmed that in the absence of AnkX, *Legionella* cannot efficiently escape phagosome maturation (Figure 5C). Moreover, we showed that phosphocholination played a critical role in *Legionella*'s ability to escape phagosome maturation. Given that amoebae are the natural host of *Legionella*, targeting of Rab35 to modulate phagosome maturation would be a desirable benefit for the bacterium. In human cells, where Rab35 regulates recycling of proteins involved in innate immunity, targeting of Rab35 by *Legionella* could offer an added benefit for the bacterium, and perhaps facilitate evasion of immune surveillance.

In conclusion, our data indicate that *Legionella* effectors inhibit endocytic recycling at the early stage of infection, and that the phosphocholination activity of AnkX plays a significant role in targeting this membrane transport pathway. Overall, our study reveals the biological relevance of AnkX-catalyzed phosphocholination during infection of human macrophages and provides new insight into subversion of membrane transport pathways by *Legionella* effectors. A complete perspective of how *Legionella* effectors target endocytic recycling merits further investigation and promises to reveal important details about survival of *Legionella* and other intracellular pathogens.

## Materials and methods

### Strains, tissue culture cells, and media

*L. pneumophila* strains Lp01 (*hsdR rpsL*) and Lp01Δ*dotA* (T4SS^−^) are derivatives of *L. pneumophila* strain Philadelphia-1 (Berger and Isberg, 1993). The wild-type Lp01, Lp01Δ*ankX* mutant, Lp01Δ*dotA*, and the pJB1806-*ankX* plasmid were a generous gift from Dr. Craig Roy. All bacterial strains used in this study are listed in Supplementary Table 1. The pJB1806-*ankX*_*H*229*A*_ shuttle vector was generated by quick-change mutagenesis using oligonucleotides listed in Supplementary Table 2 using a standard protocol. *Legionella* strains were grown in AYE liquid media or on CYE solid media and maintained as described (Feeley et al., 1979). The pJB1806-*ankX* and pJB1806-*ankX*_*H*229*A*_ shuttle vectors were transformed into the Lp01Δ*ankX* strain to complement the deletion strain with catalytically active or inactive AnkX, respectively. Bacterial growth media was supplemented with 25 μg/ml chloramphenicol to maintain pJB1806 plasmids in the Lp01Δ*ankX* strain and 1 mM IPTG was added to the medium to induce gene expression. HeLa tissue culture cells were maintained in RPMI 1640 medium supplemented with 2 mM L-glutamine and 10% FBS, and incubated in 5% CO_2_ at 37°C. COS-1 and HEK293T cells were grown in DMEM medium supplemented with 2 mM L-glutamine supplemented with 10% FBS. U937 cells were grown in RPMI 1640 medium supplemented with 10% FBS and were differentiated by supplementing the medium with 10 ng/ml 12-O -tetradecanoylphorbol-13-acetate (TPA) for 16 h.

### Antibodies and plasmids

Antibodies were purchased from BioLegend (MHC-I, clone w6/32 IgG2a), ThermoFisher Scientific (Goat anti-Rat Alexa 350, A-21093; Goat anti-Rat Texas Red, T-6392; Goat anti-Mouse Alexa 488, A-11001; Mouse monoclonal anti-GST, MA4-004; Rabbit polyclonal anti-mCherry, Pa5-34974, HRP-conjugated anti-mouse antibody, NA931), Abcam (Rabbit polyclonal anti-LAMP-2; ab37024; Rabbit polyclonal anti-GFP antibody, ab6556), Enzo (Rabbit polyclonal Calnexin, ADI-SPA-860), ProteinTech (Mouse Monoclonal Alpha Tubulin, 66031-1-Ig), and Jackson ImmunoResearch (Rabbit IgG ChromePure, 011-000-003). The rat polyclonal anti-Legionella antibody and the rabbit polyclonal anti-AnkX used for detection of purified AnkX in Figure 3 were a kind gift from Dr. Matthias Machner (NIH). We also thank Dr. Zhao-Qing Luo (Purdue University) for the rabbit polyclonal anti-AnkX antibody used for detection of AnkX in *Legionella* lysate (Supplementary Figure 5).

Full-length *ankX* and truncations were amplified using oligonucleotides in Supplementary Table 2 and were inserted by recombination into pDONR221™. The inserted fragments were then recombined into the mammalian expression vector 362-pCS-Cherry-DEST (Addgene plasmid #13075) or the pcDNA6.2/N-EmGFP-DEST vector (ThermoFisher Scientific) using the Gateway™ cloning technology (ThermoFisher Scientific). Plasmids encoding GST-tagged AnkX variants were generated by using the pDONR221-*ankX*_1−490_ and pDONR221-*ankX*_491−949_ donor vectors to recombine *ankX* into the destination plasmid pDEST15™ (ThermoFisher Scientific). The pEGFP-C1-Rab35 plasmid was a kind gift from Dr. Julie Donaldson (NIH). Plasmids used in this study are listed in Supplementary Table 1.

### Confocal microscopy

HeLa cells grown on 12 mm cover glass (Fisherbrand™) were transiently transfected with plasmids encoding fluorescently-tagged constructs using the Lipofectamine 3000 reagent (ThermoFisher Scientific). At 14 h post-transfection cells were washed with PBS and fixed with 4% paraformaldehyde in PBS for 20 min at room temperature. To visualize the plasma membrane, cells were treated with a Wheat Germ Agglutinin Alexa Fluor 488 conjugate (ThermoFisher Scientific) at 5 μg/ml for 10 min at 37°C. Coverslips were mounted using ProLong Diamond anti-fade reagent (ThermoFisher Scientific) and image acquisition was performed using a ZEISS 710 confocal microscope, a 63× Plan-Apochromatic oil immersion objective (numerical aperture of 1.4), and the ZEN 2012 software (Carl ZEISS MicroImaging).

### Transmission electron microscopy

COS-1 cells transiently producing GFP-AnkX were washed briefly with PBS and fixed overnight with 4% paraformaldehyde and 0.5% glutaraldehyde in 0.1 M sodium cacodylate buffer pH 7.4 at 4°C. Cells were then washed with 0.1 M sodium cacodylate buffer pH 7.4, scraped, and pelleted. The cell pellet was enrobed with 4% low melting point agarose and cut into small 1–2 mm^3^ cubes. The cells were washed with 0.1 M sodium cacodylate buffer pH 7.4, dehydrated in an ascending ethanol series and infiltrated with LR White resin. Samples were embedded in gelatin capsules and polymerized by UV at 4°C for 2 days. The samples were sectioned on a Reichert-Jung Ultracut E ultramicrotome, and 60–70 nm thick sections were collected onto formvar/carbon coated 200 mesh nickel grids. *Immunogold Labeling*—Samples were blocked with 0.05 M glycine for 15 min and Aurion goat blocking solution for 30 min before being incubated on drops of anti-GFP antibody diluted to 2.8 μg/ml in Aurion 0.1% BSA-c 7.4 for 1 h. Control grids were incubated on drops of ChromePure rabbit IgG (Jackson ImmunoResearch, Cat No. 011-000-003) diluted to 2.8 μg/ml in Aurion 0.1% BSA-c 7.4. Grids were washed on six drops of Aurion 0.1% BSA-c and incubated on drops of Aurion goat anti-rabbit IgG conjugated to 10 nm gold diluted 1:20 in Aurion 0.1% BSA-c for 2 h. Grids were washed on six drops of Aurion 0.1% BSA-c, 3 drops of PBS, fixed on drops of 2% glutaraldehyde in PBS, and then washed on five drops of Nanopure water. The grids were then post-stained with 2% uranyl acetate in 50% methanol and Reynolds' lead citrate. The samples were examined with a ZEISS Libra 120 transmission electron microscope operating at 120 kV, and images were acquired with a Gatan Ultrascan 1000 CCD camera.

### Cellular fractionation

HEK293T cells producing mCherry-AnkX or -AnkX variants were scraped, washed, resuspended in PBS supplemented with protease inhibitors, and lysed by 30 passes through a 27″ gauge needle. The post-nuclear supernatant (PNS) was obtained via centrifugation at 15,000 × g for 10 min at 4°C. To obtain the cytosolic fraction, the PNS was spun at 51,000 rpm for 45 min at 4°C using a TLA-100 rotor in a Beckman ultracentrifuge. PBS was added to the pellet and spun again to wash away cytosolic contaminants. The pellet was resuspended with an equal volume of 2% NP-40 in PBS and collected as the membrane fraction. The PNS, cytosolic fraction, and membrane fraction were analyzed via SDS-PAGE and immunoblot.

### Detection and quantification of LAMP-2-positive LCVs

U937 macrophages were challenged with *Legionella* strains at a multiplicity of infection (MOI) of 50 and spun at 200 × g for 5 min and then incubated at 37°C. At 2 h post-infection cells were washed three times with PBS and fixed in 4% paraformaldehyde for 20 min. To distinguish extra- and intracellular bacteria a two-step immunostaining procedure was performed. Without macrophage permeablization, *Legionella* was detected with an anti-*Legionella* rat primary antibody (1:3,000) followed by Cascade Blue-conjugated Goat anti-Rat IgG antibody (1:1,000). Cells were then permeabilized with ice-cold methanol for 20 s and internalized bacteria were detected anti-*Legionella* rat primary (1:3,000) and Texas Red-conjugated Goat α-Rat IgG (1:1,000).

For detection of LAMP-2-positive LCVs, infected U937 cells were immunostained with anti-LAMP-2-rabbit primary antibody (1:150) washed three times with PBS and fluorescently labeled with Alexa 488-rabbit secondary antibody (1:1,000). Coverslips were mounted using ProLong Diamond anti-fade reagent (ThermoFisher Scientific). The percentage of LAMP-2-positive LCVs was determined by scoring 100 cells per coverslip with three replicates for each specific condition. The unpaired two-sided Student's *t*-test was performed with a *p*-value cut off of < 0.05 to determine where there was a statistically significant difference in localization to the LCV.

### Transferrin recycling assay

To determine efficiency of recycling, U937 cells were grown on glass coverslips in a 24-well plate and following differentiation into macrophages cells were infected with *Legionella* strains Lp01, Lp01Δ*dotA*, Lp01Δ*ankX*, Lp01Δ*ankX* harboring pBJ1806-*ankX*, and Lp01Δ*ankX* harboring pJB18060-*ankX*_H229A_ at an MOI of 50 for 1 h. Infected macrophages were then allowed to uptake fluorescently labeled human transferrin (Tf-Alexa-488; ThermoFisher Scientific) at 5 μg/ml in RPMI for 1 h. Cells were either fixed to measure the fluorescence intensity after the pulse step or they were washed with PBS containing unlabeled human transferrin (100 μg/ml) three times and then incubated further with unlabeled transferrin at 100 μg/ml in RPMI for 30 min. Cells were fixed and immunostained to identify infected cells and visualized by confocal microscopy as described above. All parameters for imaging remained consistent throughout the experiment. Using ImageJ-FIJI software (Schindelin et al., 2012, 2015) the mean fluorescence intensity of Tf-Alexa-488 in 50 infected cells from three replicates for each condition was measured. To account for variation in cell size, the mean fluorescence intensity was determined by normalizing each cell's fluorescence intensity to the total area of the cell. A one-way ANOVA was performed with a *p* < 0.01 to determine if any strains showed a significant difference in recycling. *Post-Hoc* Tukey-Kramer test was performed with a *p*-value cut off of < 0.01 in order to identify which pairs of infected cells were significantly different from each other.

### Production and purification of recombinant proteins

Recombinant HaloTag-AnkX was produced in the Single Step (KRX) competent *E. coli* strain (Promega), and AnkX was purified using the HaloTag protein purification system as previously described (Neunuebel et al., 2012). Briefly, *E. coli* cells producing HaloTag-AnkX were lysed using an LV10 microfluidizer (Microfluidics). The cell lysate was spun at 25,000 × g for 20 min, and pre-equilibrated HaloLink resin were incubated with the supernatant for 2 h at 4°C. The resin was then washed and AnkX was cleaved off the resin using His-tagged tobacco etch virus (TEV) protease (Promega) for 2 h at 4°C. HisLink resin (Promega) was used to remove the TEV protease from the supernatant.

AnkX fragments were produced as GST fusion proteins in *E. coli* BL21 (DE3) at 25°C overnight after induction with 0.5 mM isopropyl-β-dithiogalactopyranoside (IPTG). *E. coli* cells producing GST-tagged variants were harvested and resuspended in PBS supplemented with 1 mM MgCl_2_ and 1 mM β-mercaptoethanol (PBS-MM) followed by lysis using the LV10 microfluidizer (Microfluidics). The cell lysate was centrifuged at 24,000 × g for 35 min, and the supernatant was incubated with pre-equilibrated Glutathione Sepharose 4B (GE Healthcare) for 2 h at 4°C. The resin was washed three times with PBS-MM, and proteins were eluted with 50 mM Tris-HCl (pH 8) containing 10 mM reduced glutathione (Sigma). Glutathione was removed by passing the eluate through a Zeba column (ThermoFisher Scientific).

### Protein-lipid overlay assay

Protein-lipid overlay assays were performed using commercially available phosphoinositide strips (Echelon Biosciences Inc.). Nitrocellulose membranes pre-spotted with different phospholipids were blocked with 3% non-fat milk in PBST [PBS and 0.1% Tween-20 (v/v) pH 7.5] for 1 h at room temperature. The blocked membranes were incubated with AnkX, GST-AnkX1-490, or GST-AnkX491-949 (0.5 μg/ml in blocking buffer) overnight at 4°C. Protein binding to lipids was visualized with an anti-GST antibody (1:2,000) or an anti-AnkX antibody, and an HRP-conjugated anti-mouse antibody (1:5,000) using a ChemiDoc Touch Imaging system (Bio-Rad).

### MHC-I internalization assay

HeLa cells were grown on cover slips and transiently transfected with mCherry-AnkX using Lipofectamine 2000 (ThermoFisher Scientific) reagent for 16 h. Following transfection cells were incubated with an anti-MHC-I antibody conjugated to Alexa 488 (1:100) for 30 min at 37°C and 5%CO_2_. After internalization, cells were rinsed twice with PBS and washed with acid (0.5% acetic acid, 0.5 M NaCl, pH 3.0) for 20 s to remove remaining surface-bound antibody. The acid stripping was followed by a PBS wash and final rinse with RPMI. Cells were fixed with 4% paraformaldehyde in PBS for 20 min at room temperature and visualized by confocal microscopy.

## Author contributions

MRN conceived the project, designed the experiments, performed the Rab35 and MHC-I co-localization experiments, and wrote the manuscript. SL and CP purified proteins and performed the protein-lipid over assays. RRN contributed to the cellular fractionation assays and analysis of the data. SCA and BPR performed the rest of the experiments, analyzed the data, and wrote the manuscript.

### Conflict of interest statement

The authors declare that the research was conducted in the absence of any commercial or financial relationships that could be construed as a potential conflict of interest.
