# Supplementary material for: Legionella Effector AnkX Disrupts Host Cell Endocytic Recycling in a Phosphocholination-Dependent Manner
Source: Front Cell Infect Microbiol. 2017 Sep 8;7:397. doi: 10.3389/fcimb.2017.00397 (PMC5596087; doi:10.3389/fcimb.2017.00397)
Supplement: Supplementary file 1 [file DataSheet1.docx]

Supplementary Material

*Legionella* effector AnkX disrupts host cell endocytic recycling in a

phosphocholination-dependent manner

Samual C. Allgood†, Barbara P. Romero Dueñas†, Rebecca R. Noll, Colleen Pike, Sean Lein, M. Ramona Neunuebel*

*** Correspondence:** M. Ramona Neunuebel: neunr@udel.edu

†S.C.A and B.P.R. contributed equally to this work

# Supplementary Tables

**Supplementary Table 1.** Microbial strains and plasmids used in this study

| **Strain or plasmid** | **Relevant features** | **Source or reference** |
| --- | --- | --- |
| ***E. coli* strains** |  |  |
| GC5 | F− *ϕ80lacZΔM15 Δ(lacZYA-argF)U169 recA1 endA1 hsdR17(rK− mK+) phoA supE44 thi-1 gyrA96 relA1λ−tonA* | ThermoFisher Scientific |
| KRX | [F´, *tra*D36, Δ*omp*P, *pro*A^+^B^+^, *lac*Iq, Δ(*lac*Z)M15] Δ*omp*T, *end*A1, *rec*A1, *gyr*A96 (Nal^r^), *thi*-1, *hsd*R17 (r_k_^–^, m_k_^+^), e14^–^ (McrA^–^), *rel*A1, *sup*E44, Δ(*lac*-*pro*AB), Δ(*rha*BAD)::T7 RNA polymerase | Promega |
| BL21(DE3) | F^−^*ompT hsdS_B_*(r_B_^−^ m_B_^−^) *gal dcm*(DE3) | ThermoFisher Scientific |
| ***L. pneumophila* strains** |  |  |
| Lp01 | Philadelphia-1, serogroup 1, salt sensitive, restriction deficient; Sm^r^ | (Berger and Isberg 1993) |
| Lp01 ∆*dotA* | Philadelphia-1, serogroup 1, salt sensitive, restriction deficient, lacking *dotA*; Sm^r^ | (Roy and Isberg 1997) |
| Lp01 ∆*ankX* | Philadelphia-1, serogroup 1, salt sensitive, restriction deficient, lacking *ankX*; Sm^r^ | (Mukherjee *et al.,* 2011) |
| Lp01 ∆*ankX-*p*ankX* | Philadelphia-1, serogroup 1, salt sensitive, restriction deficient, lacking *ankX* and carrying pJB1806-*ankX*; Sm^r^ , Cm^r^ | (Mukherjee *et al.,* 2011) |
| Lp01 ∆*ankX-*p*ankX_H229A_* | Philadelphia-1, serogroup 1, salt sensitive, restriction deficient, lacking *ankX* and carrying pJB1806-*ankX_H229A_*; Sm^r^, Cm^r^ | This study |
| **Plasmids** |  |  |
| pcDNA6.2N/EmGFP-DEST | Encodes GFP; Cm^r^, Amp^r^ | ThermoFisher Scientific |
| pcDNA6.2N/EmGFP-DEST-*ankX* | Encoding GFP-tagged AnkX ; Amp^r^ | ThermoFisher Scientific |
| 362 pCS-Cherry-DEST | Encodes mCherry; Cam^r^, Amp^r^ | Addgene |
| 362 pCS-Cherry-DEST-*ankX* | Encoding mCherry-tagged AnkX ; Amp^r^ | This study |
| 362 pCS-Cherry-DEST-*ankX*_1-140_ | Encoding mCherry-tagged AnkX fragment (aa 1-140); Amp^r^ | This study |
| 362 pCS-Cherry-DEST-*ankX*_491-809_ | Encoding mCherry-tagged AnkX fragment (aa 491-809); Amp^r^ | This study |
| 362 pCS-Cherry-DEST-*ankX*_810-949_ | Encoding mCherry-tagged AnkX fragment (aa 810-949); Amp^r^ | This study |
| 362 pCS-Cherry-DEST-*ankX*_491-949_ | Encoding mCherry-tagged AnkX fragment (aa 491-949); Amp^r^ | This study |
| 362 pCS-Cherry-DEST-*ankX*_688-949_ | Encoding mCherry-tagged AnkX fragment (aa 688-949); Amp^r^ | This study |
| 362 pCS-Cherry-DEST-*ankX*_721-949_ | Encoding mCherry-tagged AnkX fragment (aa 721-949); Amp^r^ | This study |
| pFN22K HaloTag® CMV*d1* Flexi® | Encodes HaloTag; Kan^r^ | Promega |
| pFN22K HaloTag CMV*d1* Flexi-*ankX* | Encodes HaloTag-AnkX; Kan^r^ | This study |
| pJB1806-*ankX* | pJB1806 carrying the ankX gene; Amp^r^ , Cm^r^ | (Mukherjee *et al.,* 2011) |
| pJB1806- *ankX_H229A_* | pJB1806 carrying the *ankX_H229A_* gene; Amp^r^ , Cm^r^ | This study |
| pDONR221 | Gateway entry vector; Kan^r^ | ThermoFisher Scientific |
| pDONR221-*ankX_1-490_* | pDONR221 carrying *ankX_1-490_*; Kan^r^ | This study |
| pDONR221-*ankX_491-949_* | pDONR221 carrying *ankX_491-949_*; Kan^r^ | This study |
| pDONR221-*ankX_491-809_* | pDONR221 carrying *ankX_491-809_*; Kan^r^ | This study |
| pDONR221-*ankX_810-949_* | pDONR221 carrying *ankX_810-949_*; Kan^r^ | This study |
| pDEST15 | Gateway destination vector encoding GST; Amp^r^ | ThermoFisher Scientific |
| pDEST15-*ankX_1-490_* | Encoding GST-tagged AnkX (aa 1-490); Amp^r^ | This study |
| pDEST15-*ankX_491-949_* | Encoding GST-tagged AnkX (aa 491-949); Amp^r^ | This study |

Abbreviations: Kan^r^, kanamycin resistance; Amp^r^, ampicillin resistance; Cm^r^, chloramphenicol resistance, Sm^r^, streptomycin resistance.

**Supplementary Table 2.** List of oligonucleotides used for this study.

| **Name** | **Sequence 5’ to 3’ direction** |
| --- | --- |
| Fw-attB1-AnkX-1 | GGGGACAAGTTTGTACAAAAAAGCAGGCTTCATGTTGGTAAAAATTATGCCAAAT |
| Fw-attB1-AnkX-491 | GGGGACAAGTTTGTACAAAAAAGCAGGCTTCATGCAGATTAATGAACCGGATAAC |
| Rv-attB2-AnkX-949 | GGGGACCACTTTGTACAAGAAAGCTGGGTCTTACCATTTTAATTTCAA |
| Rv-attB2-AnkX-809 | GGGGACCACTTTGTACAAGAAAGCTGGGTCCTAGTTTTTAAGAAATTT |
| Fw-attB1-AnkX-688 | GGGGACAACTTTGTACAAAAAAGCAGGCTTCATGGAAGACAGGCTGGGAAATAA |
| Fw-attB1-AnkX-721 | GGGGACAAGTTTGTACAAAAAAGCAGGCTTCATGAAAAGAAACTCTGAAAGAAG |
| Fw-attB1-AnkX-810 | GGGGACAAGTTTGTACAAAAAAGCAGGCTTCATGTCCGATGCCAATTCTATTTTG |
| Fw-SgfI-AnkX | GATCGCGATCGCCATGTTGGTAAAAATTATGCCAAATC |
| Rv-PmeI-AnkX | GATCGTTTAAACCCATTTTAATTTCAAGGATG |
| Fw-AnkX H229A | CATATTCGAATGTACGAAGTATTAGCCCCTTTTCGA |
| Rv-AnkX H229A | TCGAAAAGGGGCTAATACTTCGTACATTCGAATATG |

#
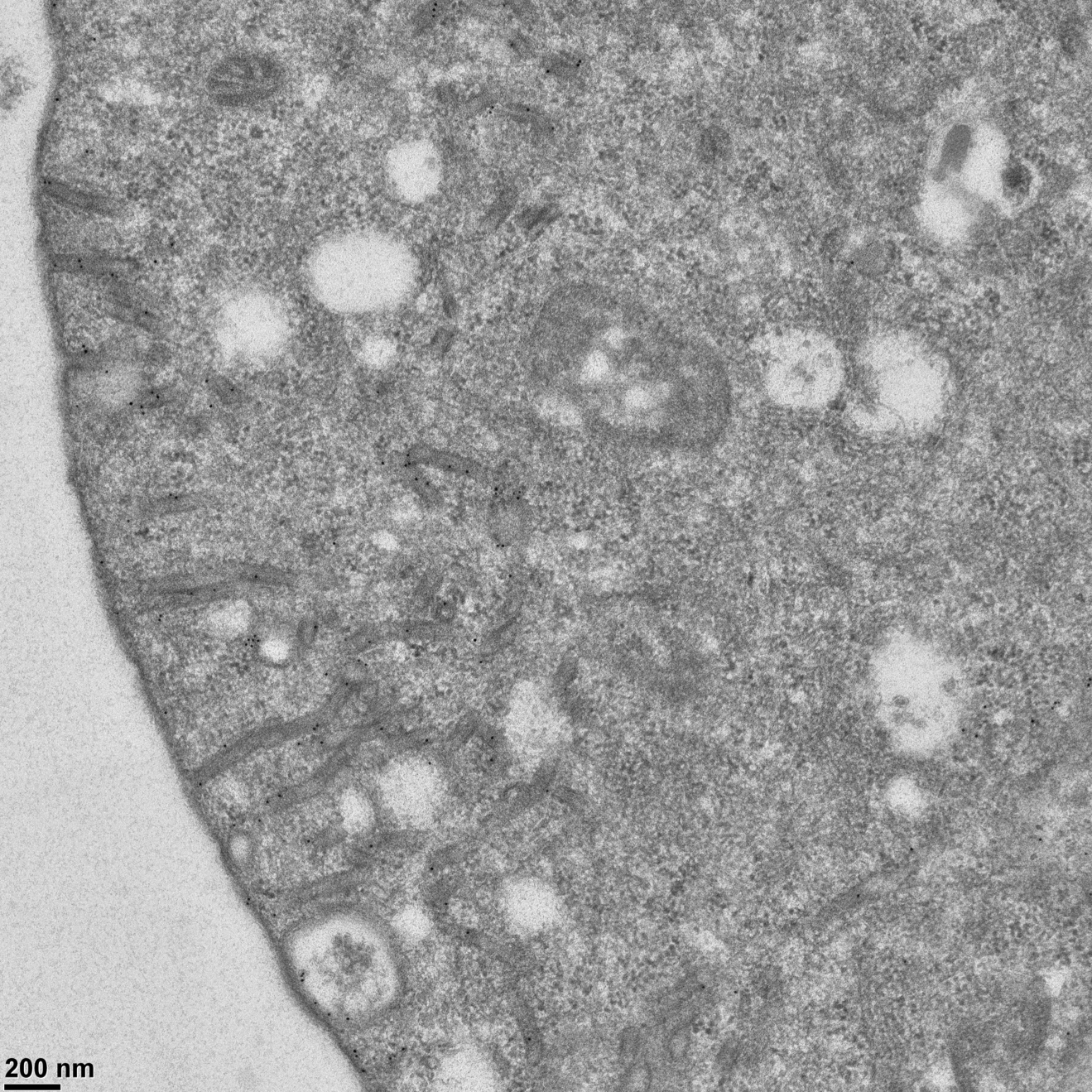
Supplementary Figures

**Supplementary Figure 1. Overview of GFP-AnkX localization.** Immunogold TEM micrograph of a COS-1 cell ectopically producing GFP-AnkX using rabbit polyclonal anti-GFP.


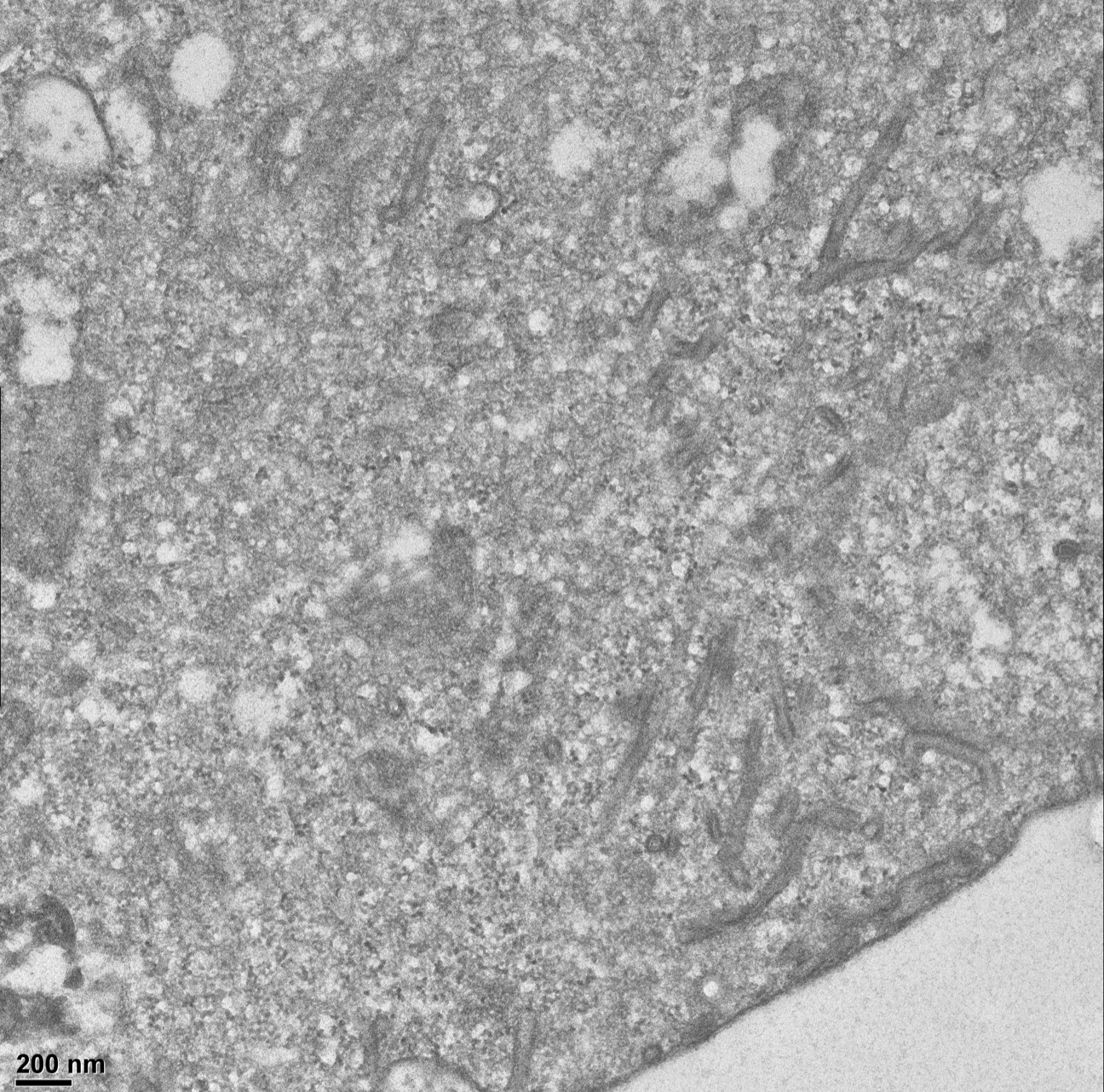


**Supplementary Figure 2.** **Control Immunogold TEM micrograph.** COS-1 cells ectopically producing GFP-AnkX were incubated with anti-rabbit IgG.

**Supplementary Figure 3.** **Purified full-length and truncated AnkX.** Purified proteins – AnkX (109 KDa), GST-AnkX1-490 (81KDa) and GST-AnkX491-949 (78KDa) – were separated by SDS-PAGE electrophoresis on a 4-15% TGX stain-free gel.


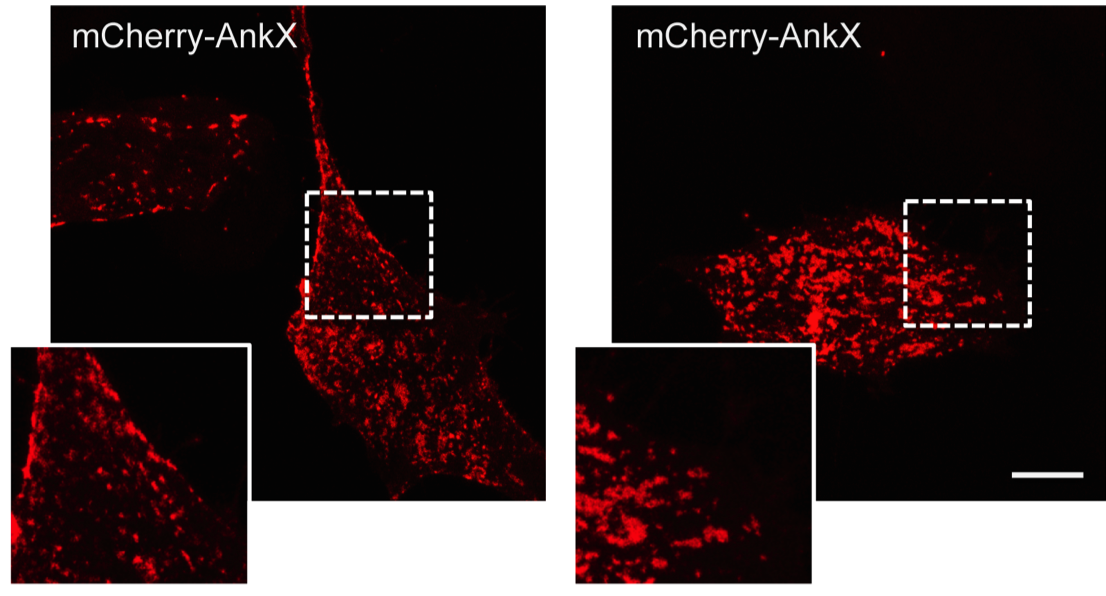


**Supplementary Figure 4. Representative images of mCherry-AnkX localization in HeLa cells.** HeLa cells transfected (for 12 hours) with a plasmid encoding mCherry-AnkX were fixed and visualized by confocal microscopy. Micrographs are maximum intensity projections of a confocal z-stack. In the left panel mCherry-AnkX localizes non-uniformly along the plasma membrane and has a patchy distribution in the rest of the cell. In the right panel localization at the plasma membrane is not apparent (seen in 5% of the transfected cells). Insets show a magnified view of an area of interest.

*ΔankX*-p*ankX*

*ΔankX*-p*ankX_H229A_*

100 kDa

α-AnkX


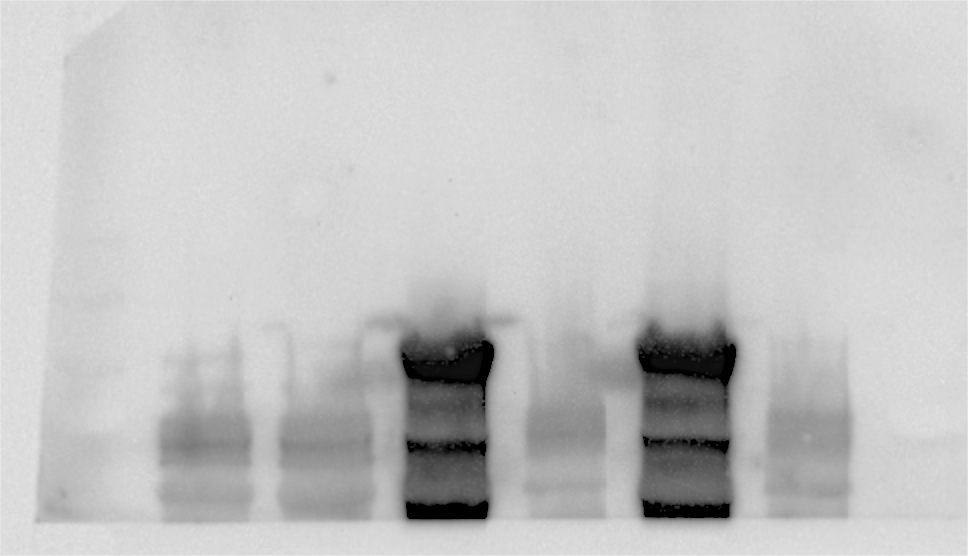

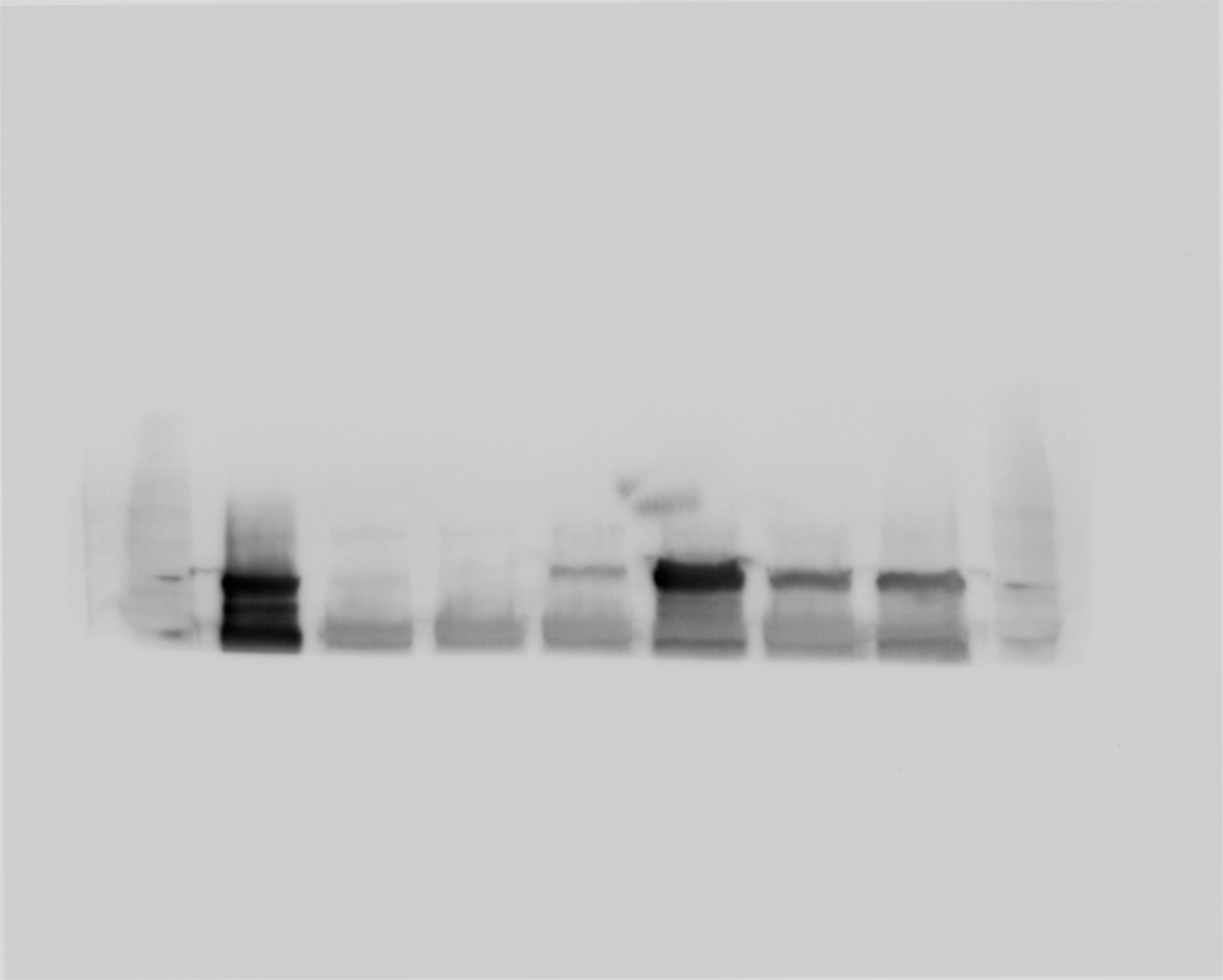

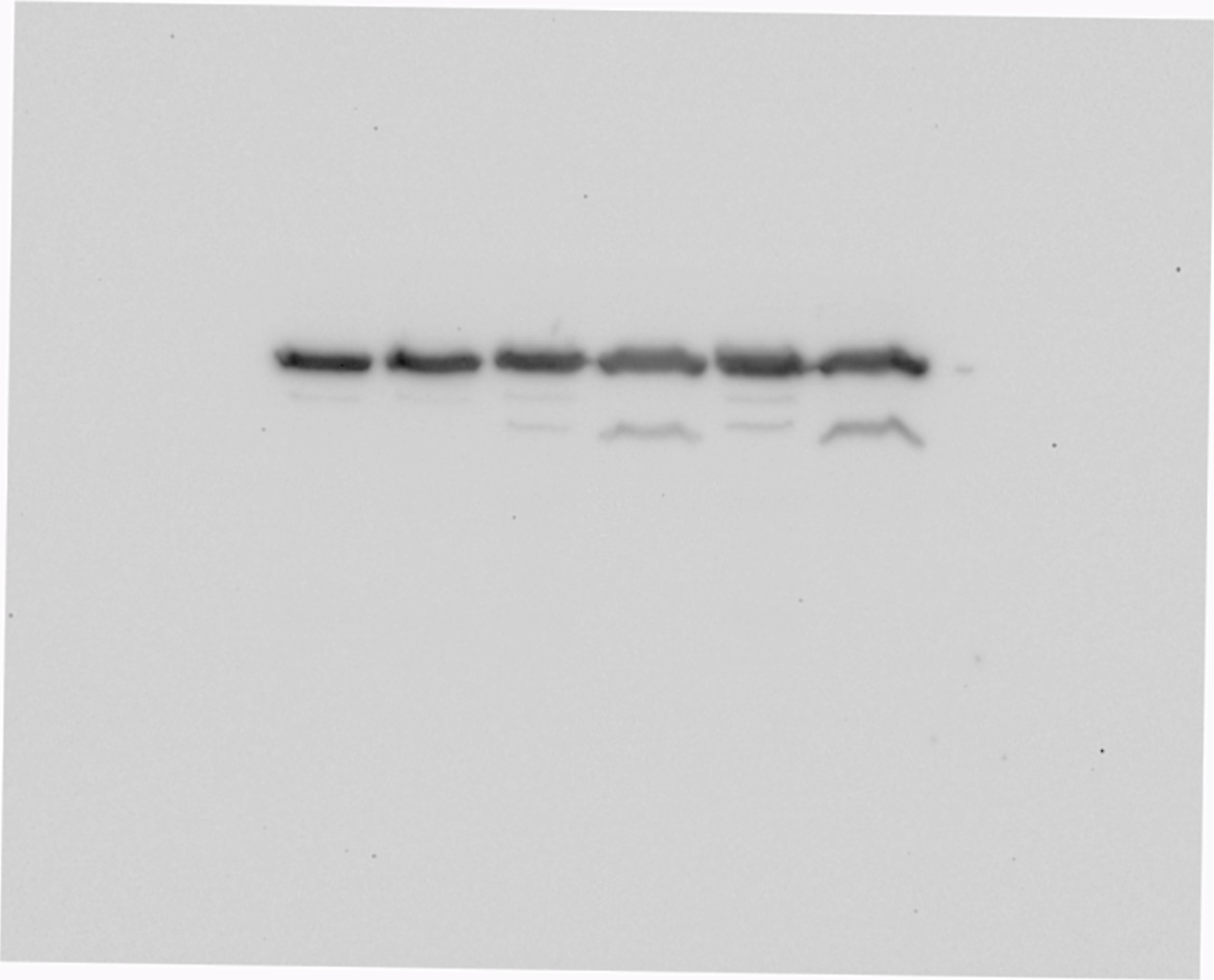

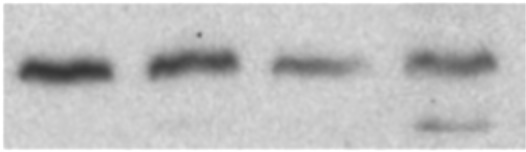


α-ICDH

IPTG

+

-

+

-

IPTG

*ΔankX*

Lp01

His-AnkX

**Supplementary Figure 5. Levels of AnkX produced by complemented Lp01*∆ankX* strains.** AnkX levels in *Legionella* strains Lp01, *∆ankX*, *∆ankX* complemented with pJB1806-*ankX* or pJB1806-*ankX_H229A_* was analyzed via western blot. Lysates (2.56×10^8^ cells) were prepared by boiling for 10 minutes in 30 μl 1X  Laemmli buffer and then cleared by centrifugation at 11,000×g for 1 minute. The lysates were then run alongside 3 μg of purified His-AnkX on a 12% SDS-PAGE gel and transferred to a PVDF membrane. ICDH was used as a loading control

**Supplementary Figure 6. U937 cells infected with different *Legionella* strains have similar levels of internalized transferrin.** At 1 hour post-infection, U937 cells were incubated with transferrin-Alexa 488 for 60 minutes, then briefly washed with PBS, fixed, immunostained with anti-*Legionella* antibodies, and visualized by confocal microscopy. The graph displays the amount of fluorescence retained by infected U937 cells. The mean and standard error of the mean from three independent experiments are indicated for each condition. Statistical significance was determined by one-way ANOVA followed by Tukey Kramer post hoc test.

**References**

Berger, K. H. and R. R. Isberg (1993). Two distinct defects in intracellular growth complemented by a single genetic locus in Legionella pneumophila. *Mol Microbiol* 7(1): 7-19.

Mukherjee, S., X. Liu, K. Arasaki, J. McDonough, J. E. Galan and C. R. Roy (2011). Modulation of Rab GTPase function by a protein phosphocholine transferase. *Nature*.

Roy, C. R. and R. R. Isberg (1997). Topology of Legionella pneumophila DotA: an inner membrane protein required for replication in macrophages. *Infect Immun* 65(2): 571-578.
